# Research on Disparities in Primary Health Care in Rural versus Urban Areas: Select Perspectives

**DOI:** 10.3390/ijerph19127110

**Published:** 2022-06-10

**Authors:** Jayasree Basu

**Affiliations:** Independent Researcher, Rockville, MD 20857, USA; basuj181@gmail.com

## 1. Introduction

Much of the differences in health care outcomes can be attributed to the differential rates of primary health care utilization and resource allocation across population subgroups. A study highlighting these differences in rural versus urban regions, may focus on populations stratified by socioeconomic factors such as age, gender, and income, as well as other factors affecting their access to care. The appropriateness of these issues remains particularly significant in the context of a developing country with a large rural population and significant disparities characterizing the health care system. A comparative analysis between rural and urban regions is also important to highlight the differences in these socioeconomic factors associated with primary care access.

This perspective provides a protocol for studying disparities, particularly with reference to the patterns of primary health care utilization in economies with a large rural span. The paper is organized in a way to provide a comprehensive framework of a prospective study protocol by highlighting its contextual and organizational perspective, as well as incorporating a conceptual model to address specific research questions, along with a section on research design and data needs. A study on urban-rural disparities in primary care is particularly important in view of the growing need to reallocate scarce health care resources resulting from the outbreak of the COVID-19 pandemic, which disproportionately affected villages in recent months [1].

## 2. Background and Motivation

The World Health Organization’s *World Health Report 2008* [2] recognized the important roles and value of primary care. Key facts relative to the current state of the rural healthcare system have been highlighted in reports by the National Rural Health Mission [3] and by the United Nations [4]. The reports indicate that rural areas are disproportionately lacking in health care resources compared to urban areas. Thus, there is a significant need for more research, evaluation, and policy mandates to improve the rural primary health care system.

Many of the inequities in health result from a wide range of social, economic, and political circumstances or factors that differentially affect the distribution of health within a population. Some of these factors have been identified in the context of the general population of a developing country [5] but have not been specifically addressed for its rural areas. Some of the issues on rural primary care in more developed economies such as the USA have been addressed in previous research [6,7,8,9,10,11,12].

Studying disparities in primary care is imperative since research [13,14] has shown that primary care may reduce the negative health effects of economic inequality on health and mortality, especially in areas where income inequality is the greatest. For example, according to a report by the United Nations, 75% of the health infrastructure in a country like India–including doctors and specialists, and other health resources–is concentrated in urban areas where only 27% of India’s population lives. The rural population of India is around 716 million people (72%) and yet there is a chronic lack of proper medical facilities [4]. Previous work suggests that rural health care in India faces a crisis unmatched by any other sector of the economy [15,16].

The proposed study is highly significant because primary care is a cost-effective way of preventing hospital admissions. Hospital admissions are costly, less affordable for rural residents, and may cause a delay in treatment as well as death [17,18]. Research [9] has shown that the hospitalizations for certain conditions such as Hypertension, Diabetes, Cellulitis, Convulsions, Gastroenteritis, Hypoglycemia, kidney infection, Asthma, and Dehydration, may be avoided if clinicians effectively diagnose, treat, and educate patients, and if patients actively participate in their care and adopt healthy lifestyle behaviors. Emerging studies clearly show that there is a direct correlation between greater accessibility to primary care, particularly to family doctors and interprofessional teams, and better health of the citizens within a geographic area [19]. These issues continue to be very important during and beyond the post-COVID era.

## 3. Conceptual Model and Research Questions

An examination of the primary care system should begin by looking at both supply and demand issues in health care as it currently exists. On the supply side, one needs to evaluate health care resource supplies in rural versus urban regions, some of which have been documented in reports described above (e.g., 4). Some key supply indicators could include the availability of primary care clinics and hospital beds, distance to nearby clinics and hospitals, and transportation and transfer of severe cases to urban hospitals. Equally important as the supply side, on the demand side one should seek to understand behavioral differences associated with healthcare use. In this context, important work conducted by Aday and Anderson [20] and by Anderson [21] should be highlighted. Their behavioral models of access to care include several factors including predisposing factors such as age, race, and gender of patients, enabling factors such as socioeconomic status and affordability of care, as well as patients’ need levels such as comorbidities and severity of illness.

One of the predisposing factors, the patient’s gender, was often highlighted in the context of developing countries with a large rural population. Previous research has shown that health care seeking behaviors are different across women and men, particularly in rural areas. This is because women, particularly female children, often get preventive care less frequently than men, or may face greater delays in getting appropriate primary care in rural districts of some regions [22,23,24,25,26]. One outcome of this disparity in access could be that the lack of appropriate primary care may result in rural women being admitted to hospitals more frequently with conditions that can be prevented in the outpatient care unit or care provided in physicians’ offices. Another outcome could be that rural women will be hospitalized with higher complexity than rural men, which could also result in higher chances of disability and death [27]. The effect of gender bias on health care outcome is a significant issue that needs further attention in the context of a rural economy. An in-depth assessment could be performed on gender disparities and bias as it affects the health care outcomes of patients, and overwhelmingly affects essential health care [28].

The issue of gender disparities has also been reflected in recent statistics on COVID-19 incidence and mortality. While global data indicate higher COVID-19 case fatality rates among men than women, several countries, such as India, reported such rates to be higher for women than men. As of 30 September 2020, India had more than 6.4 million recorded COVID-19 cases, while case fatality rates among men and women were, respectively, 2.9 percent and 3.3 percent [29]. Several factors have been cited as possible causes including the unequal burden of unpaid labor borne by women, their lack of attention to health and nutrition, as well as barriers to primary care access [30].

Reducing gender disparities in primary health care seeking behavior of rural residents will result in substantial improvement in the quality of life and reduce costs to families. Using a hospital-based retrospective analysis, previous research identified the maternal mortality ratio (MMR) to be very high among women in some rural economies, and the researchers concluded that most maternal deaths are preventable by intervention at the appropriate time [27]. Gender disparities in primary health care also have been clearly validated and documented in the Indian context [31].

Another such important factor that has been studied in the rural-urban context is the availability of local care and out-of-area travel patterns of rural residents to receive the needed care. The spatial accessibility in care is an important enabling factor causing rural-urban disparity, as suggested in several studies, and is deemed as a significant predictor of access, especially in countries with a large rural sector. Studies, however, also suggest that the travel patterns of rural residents could vary depending on the illness severity, affordability of care such as income and insurance status, as well as community resources, among others [7,8,11]. Gender disparities along with travel impediments, could have significant adverse effects on health care outcomes in the countries characterized by a large rural population.

Based on the conceptual framework and several key issues discussed above, the following are a few questions that may deserve further investigation across the healthcare supply and demand domains of rural versus urban residents:

- Do rural women face greater delays in accessing primary health care e.g., immunizations, routine annual visits, etc.?
- Are rural patients with the same socioeconomic status as urban citizens likely to receive comparable primary health care, given no resource constraints? In other words, is there a cultural aspect of delaying care for rural patients?
- What role does lack of rural resources (e.g., primary health care clinics) play in hospitalization patterns of rural residents in general, and rural women in particular?
- What is the role of travel distance to needed care that could affect access to care of rural patients more adversely than of urban patients?
- Are women and the elderly or poor less likely to travel longer distances to nearby urban hospitals?
- What are the most common types of conditions ignored/self-treated by rural residents, which later result in hospital admissions for preventable chronic diseases? Are these different from the types of conditions in urban areas?

## 4. Analytical Strategy

A particularly effective strategy for a large-scale study to address these issues should ideally use a mixed method analysis, in which quantitative data should be supplemented by case studies and focus group discussions within rural and urban parts of regions. The computational analysis should use the data collected through published and unpublished sources including the World Health Organization and Census or other local sources. As the data permits, statistical methods, such as ANOVA and hypothesis testing, should be used to study disparities in the socioeconomic factors across rural and urban areas. The qualitative analytic method is expected to supplement the analysis where data will be unavailable and will involve case studies as well as focus group discussions with local scientists, public health officials, primary care clinic operators, and physicians. The details of the study will be fine-tuned as one discovers the available resources and limitations.

The specific scope of the project may be determined in consultation with local scientists. As needed, the study may focus on specific regions of the country, where shortages in primary care resources and hospital beds have been reported for rural areas. For comparison purposes, an additional region or state can be selected where primary care resources are better available.

## 5. Conclusions

Many of the inequities in health result from a wide range of social, economic, and political circumstances or factors that differentially affect the distribution of health within a population. The article outlines the background and motivation for studies addressing rural-urban disparities, especially in relation to the utilization of primary care services. In addition, the article also summarizes a conceptual framework for prospective studies. Derived from this conceptual framework, several key research questions are highlighted that can be studied either singularly or as a group, by specific regions or by local areas, by individual conditions or as a set of conditions, and in the context of a developed or a developing country.

## Data Availability

Not applicable.
